# Exposure to public natural space as a protective factor for emotional well-being among young people in Canada

**DOI:** 10.1186/1471-2458-13-407

**Published:** 2013-04-29

**Authors:** Quynh Huynh, Wendy Craig, Ian Janssen, William Pickett

**Affiliations:** 1 Department of Community Health and Epidemiology, Queen’s University, Kingston, Canada; 2 Department of Psychology, Queen’s University, Kingston, Canada; 3 School of Kinesiology and Health Studies, Queen’s University, Kingston, Canada; 4 Department of Emergency Medicine, Queen’s University, Kingston, Canada

**Keywords:** Adolescence, Determinants of health, Emotional well-being, Green space, Nature, Pediatrics

## Abstract

**Background:**

Positive emotional well-being is fundamentally important to general health status, and is linked to many favorable health outcomes. There is societal interest in understanding determinants of emotional well-being in adolescence, and the natural environment represents one potential determinant. Psychological and experimental research have each shown links between exposure to nature and both stress reduction and attention restoration. Some population studies have suggested positive effects of green space on various indicators of health. However, there are limited large-scale epidemiological studies assessing this relationship, specifically for populations of young people and in the Canadian context. The objective of this study was to examine the relationship between exposure to public natural space and positive emotional well-being among young adolescent Canadians.

**Methods:**

This cross-sectional study was based upon the Canadian 2009/10 Health Behaviour in School-aged Children Survey with linked geographic information system (GIS) data. Following exclusions, the sample included 17 249 (grades 6 to 10, mostly ages 11 to 16) students from 317 schools. Features of the natural environment were extracted using GIS within a 5 km radius circular buffer surrounding each school. Multilevel logistic regression was used to examine the relationship between the presence of public natural space (features include green and blue spaces such as parks, wooded areas, and water bodies) and students’ reports of positive emotional well-being, while controlling for salient covariates and the clustered nature of the data.

**Results:**

Over half of Canadian youth reported positive emotional well-being (58.5% among boys and 51.6% among girls). Relationships between measures of natural space and positive emotional well-being were weak and lacked consistency overall, but modest protective effects were observed in small cities. Positive emotional well-being was more strongly associated with other factors including demographic characteristics, family affluence, and perceptions of neighbourhood surroundings.

**Conclusion:**

Exposure to natural space in youth’s immediate living environment may not be a leading determinant of their emotional well-being. The relationship between natural space and positive emotional well-being may be context specific, and thus different for Canadian youth compared to adult populations and those studied in other nations. Factors of the individual context were stronger potential determinants.

## Background

Positive emotional well-being is defined as an awareness of one’s well-being with a positive outlook on life [1]. It is an important aspect of overall health. Beyond the absence of distress, it encompasses the presence of positive affective states. Attention to positive psychology helps to build “human strengths and virtues including hope, wisdom, creativity, courage, spirituality, responsibility, perseverance, and satisfaction” [2]. Among young people, positive emotional well-being contributes to the development of intrapersonal and interpersonal relationships [2]. It is also associated with higher academic and vocational performance [3], and mediates the impact of stressful life events [4] and problem behaviors [1]. A lack of positive emotional well-being is an indicator of physical and mental health problems [3-5]. Experiences during childhood and adolescence have long-term implications [6,7], and positive emotional well-being earlier in life influences health trajectories into adulthood. Therefore, it is important to understand emotional well-being during critical developmental stages, such as adolescence.

According to the Public Health Agency of Canada, in 2006, 56% of Canadian boys and 48% of girls in grades 9 and 10 experienced high levels of life satisfaction, one indicator of emotional well-being [8]. The World Health Organization estimated that in 2009, 20% of the world’s population of children and adolescents had mental disorders or problems [9]. Low rates of positive emotional well-being and an increasing prevalence of early mental disorders suggest the need for a greater understanding of determinants of emotional well-being, and any protective effects of efforts to promote positive emotional well-being among this age group.

On a broad scale, determinants of health include both individual and contextual factors [10]. One potential contextual determinant of emotional well-being is the natural environment. As area-level interventions emerge as important theoretical and policy-driven strategies to address population health issues [11-13], there is increased interest in examining the relationship between nature and health. Such evidence can be used to modify and build communities that take advantage of natural space in order to promote positive well-being for the general population. Theories underlying this relationship are based upon stress reduction and attention restoration [14]; people have nature-based coping strategies, and thus natural space helps to facilitate restoration from stress and fatigue. Other explanations suggest that natural settings offer added opportunities for explorations and interactions, and activities that promote greater levels of well-being [15-18].

Although there is a common belief that nature has health benefits, this is not clearly supported by empirical evidence, particularly at the population level. Much of the existing literature is descriptive [19-21], and few population studies have quantified this relationship. A body of psychological research has shown that exposure to green features, such as trees and gardens, positively influences stress reduction and attention restoration [14]. This research has been limited to controlled settings, and has focused on proximal measures such as images of nature [22], views from windows [23,24], and access to nearby gardens [25]. Others have demonstrated added benefits for activities performed in natural settings, particularly focusing on physical activities [26].

Recently, there have been efforts to examine the relationship between nature and health at the population level. Large epidemiological studies conducted with the general public, although consisting mostly of adults, have concluded that residents of neighborhoods with high proportions of green space experience better perceived health [27,28], greater physical activity [29,30], lower morbidity [31], and higher life longevity [32]. Evidence from such studies is inconclusive; although most studies support a beneficial effect, the effects tend to be weak [30-32]. Systematic reviews and meta-analyses have similarly concluded that a weak relationship exists [19,26].

The lack of consensus in the current literature may be due in part to variable definitions: most studies have used green space to represent the natural environment, but inclusion criteria vary [19,26]. Few studies have used objective measures to quantify exposures to green space and sources of natural space [27,28]. Further, green space may not be the only contributors to the relationship between nature and well-being. Exposure to water areas, known as blue space, has also been linked to stress reduction and attention restoration [33]. A few studies that have considered effects of exposure to blue space had reported added benefits: for example, improvements in self-esteem and mood after exercise were greater in the presence of water compared to green space alone [34]. Although exposure to water in the natural environment has been posited as a theoretical mechanism for relations with well-being, most studies have not measured blue space. Additionally, the outcome of health has been conceptualized differently across studies, ranging from general mental and physical health statuses under which emotional well-being may encompass [27,28] to specific emotional states, clinically-diagnosed mental illnesses, and mortality.

Few population health studies have examined the effects of natural space on health among youth populations. Although small-scale experimental studies have examined the effects of nature on children’s behaviors, most large-scale studies have been conducted in adult populations [35-37]. Findings from the few analyses that have included youth have been inconsistent due to the use of varying health outcomes and different age groups to define youth. According to de Vries *et al.*, green living environments were not associated with reported health symptoms, perceived general health, or perceived mental health among Dutch youth less than 16 years old [38]. A later study from the same research group reported that the relationship between green space and morbidity indicators, such as lower prevalence of depression, was strongest among youth less than 12 years old [31]. In the United Kingdom, Barton and Pretty concluded that effects of green environments on self-esteem and mood were strongest in young people, with a young person defined as less than 30 years [34]. Hence, the relationship between nature and emotional well-being has not been explicitly studied, and remains unclear among youth populations.

The present study was conducted to examine the relationship between public natural space and positive emotional well-being specifically among adolescents. This is one of few large-scale studies using robust methods and objective measures of both green and blue spaces. Understanding the effects of the surrounding environment on health has important implications in creating healthy communities. However, current public policies that deal with community settings are primarily based upon evidence generated from adults [39,40]. It is important to fill this gap in understanding because environmental research in adults does not always translate to younger populations [39]. Through this study, we hoped to inform decision makers on the priority of public natural space in societal efforts to promote positive emotional well-being among young people.

## Methods

This cross-sectional study was based on Canadian records from the 2009/10 Health Behaviour in School-aged Children (HBSC) Survey which collected information on demographic, behavioral, and contextual factors that influence the health of young people [41]. The individual-level HBSC records were linked to area-level data from a geographic information system [42,43] within a 5 km radius circular buffer of schools to compile objective measures describing surrounding public natural space. Other variables derived from the 2006 Canada Census of Population [44]. Environmental data were subsequently related to reports of emotional well-being, while controlling for covariates and accounting for the multilevel structure of the data.

### Study survey

The 2009/10 HBSC surveyed 26 078 students, grades 6 to 10 (mostly ages 11 to 16 years), within 436 schools using an established international protocol [45]. The sample was representative of the distribution of schools by size, location (province/territory), language (English/French), and school board type (public/separate). The HBSC survey provided individual-level data for positive emotional well-being and other covariates. In addition to the Student Questionnaire, the HBSC includes an Administrator Questionnaire completed by a principal or vice-principal, which provided information about each school and its neighborhood environment. This study used two area-level items to inquire about neighborhood aesthetics surrounding schools.

### Natural space

Features of the public natural environment were obtained from the CanMap RouteLogistics (version 2009.4) and Enhanced Points of Interests (version 2009.3) databases (DMTI Spatial Inc., Markham, ON). This is a cross-national geographic information system with accurately positioned geospatial data for boundaries and topographic features such as land-use classifications [42,43]. Data describing the natural environment were extracted and linked to school addresses from the HBSC using ArcGIS 9.3 software (ESRI, Redlands, CA) within a 5 km radius circular buffer of schools. The 5 km buffer acts as a proxy for the neighborhood environment in which students spend the most time, consisting of their school and home neighborhoods. Students interact with the school neighborhood during the day, and spend time in the space surrounding the school during their travels to and from school as well as during off-premise trips. Thus, this space is relevant to their health and health-related behaviors. The use of this buffer size is considered reliable for social constructs [46-48].

Features included in this extraction were: “*local parks and sport fields, provincial/territorial parks, national parks, other parks, wooded areas, campgrounds, picnic areas, golf courses, driving ranges, national wildlife and migratory areas, botanical gardens, and water bodies (such as oceans, lakes, rivers, streams)*”. This procedure resulted in 82 buffers, primarily in remote northern regions, with no green space area. These buffers were visually examined using Google Earth 6 software (Google Inc., Mountain View, CA), which suggested the presence of green space. Therefore, it was assumed that the GIS database was incomplete for these regions, and these 82 schools and their students were excluded. There were 35 buffers with no water areas. The same quality checks were employed and these buffers were included in the analysis.

This study measured public natural space in three ways: total natural space, green space, and blue space. Total natural space was the total area of the all public natural features collected within the buffer, green space included only land features, and blue space included only water features from the extraction. The percentage of total land within each buffer that consisted of total natural space, green space, and blue space was estimated, and the buffers were divided into equal quartiles based upon the distribution of values for each measure.

### Positive emotional well-being

Positive emotional well-being was measured using the Cantril ladder [49]. Students were asked to rank their current state of life on a 10-point scale, ranging from worst possible (0) to best possible (10) life (see Additional file 1). Positive emotional well-being was measured by a score of 8 and above, based upon established precedents [45,50]. Unlike measures of emotional feelings to immediate triggers, this ladder is a direct and global indicator of subjective well-being over time [45,51]. The Cantril ladder is an established research tool, often used to measure subjective well-being, life satisfaction, quality of life, and overall happiness. It has been considered well-developed in the literature with good validity and stability, and reasonable reliability [52]. The use of the ladder has been adapted for young people [45].

### Potential covariates

Potential covariates were considered *a priori* based upon associations with emotional well-being and/or their use in similar research. These variables will be discussed according to their hypothesized effects as a potential confounder and/or effect modifier (moderator), and justifications follow.

#### Confounders

Individual socio-economic status (SES) and perceived neighborhood safety were self-reported by student participants. SES has been shown to be a strong determinant of health [53], and has been identified as a common confounder in previous research [28,31,32,36]. The current analysis used the Family Affluence Scale (FAS) to represent student SES. This scale combines four items of equal weight: number of vehicles owned in family, having a bedroom to oneself, number of family vacations in the past year, and number of computers owned [54]. Perceived neighborhood safety may be influenced by physical neighborhood features, and may play a role in the neighborhood context of health [55]. This variable was based on student responses using a Likert scale (‘strongly agree’ to ‘strongly disagree’) to the statement “*it is safe for younger children to play outside during the day”*.

Potential confounders at the area level included neighborhood aesthetics, neighborhood SES, and urban/rural geographic location. Neighborhoods perceived as having better aesthetics have been associated with better self-reported mental health in adults [56]. Two questions describing neighborhood aesthetics were obtained from the HBSC Administrator Questionnaire. Each school administrator was asked to what extent there were “*garbage, litter, or broken glass in the street or road, on sidewalks, or in yards”* and *“vacant/shabby houses and buildings”* in their school’s neighborhood. Three items from the 2006 Canadian Census [44] were used to obtain a composite measure representing neighborhood SES: median household income, employment rate, and the percentage of the population with greater than high school education. Rank scores for these items were combined for each buffer, and buffers were divided into low, medium, and high tertiles of SES based on overall rank scores, as per existing precedents [47,48]. Urban/rural geographic location was hypothesized to be associated with natural space [57] and has been shown to be associated with health outcomes [58]. This study classified each buffer according to community size as defined by Statistics Canada and measured by the Census (population counts from 2001 and 2006): *rural area (<10 000 persons), small city (10 000 – 99 999 persons),* or *metropolitan area (>100 000 persons)*[59].

#### Effect modifiers (Moderators)

Age, gender, and ethnicity were each hypothesized to modify the relationship between natural space and positive emotional well-being. The relationship between nature and health has been shown to be different among different age groups [31,34,38]. With respect to gender, girls experience lower emotional well-being compared to boys, thus it is sensible to examine the relationship separately [8]. Ethnicity has also been shown to affect health status among children [60] and may modify the relationship between nature and health. For example, in a study among the Dutch population, effect estimates were different for European descendents and non-European immigrants [27]. In our study, ethnicity was self-identified by student participants, and categories were combined based on similarities in geography and/or culture. Urban/rural geographic location was also considered as a potential moderator as there has been evidence for differences in the relationship between nature and health across difference levels of urbanicity [27,38].

### Study population

**Figure 1:**
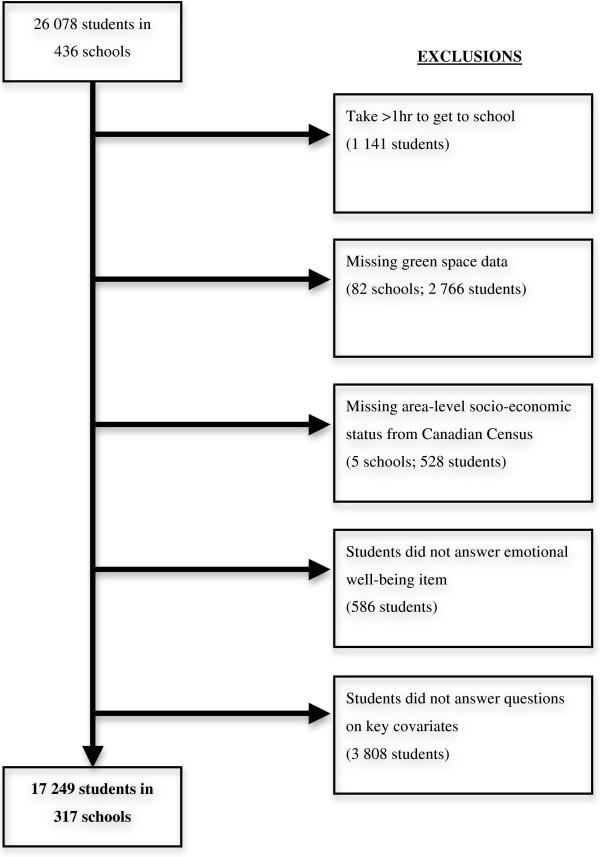
Study sample flow chart showing incremental order of exclusion.

For this analysis, students who lived beyond a 1-hour travel distance from school were excluded as the 5 km residential space surrounding school had less relevance to their living environment. Additionally, students attending the 82 schools with missing green space information were excluded. This resulted in a total of 22 171 students in 354 schools available for study. Upon removal of missing data for other key variables, the final sample was 17 249 students in 317 schools (Figure 1). There were no significant differences between those excluded and included in the analysis by most demographic characteristics (age, ethnicity, family affluence) and other potential covariates. The final sample had a slightly higher proportion of girls (by 2.9%) and a slightly lower proportion of those reporting positive emotional well-being (by 3.0%). A slightly lower proportion of land area in the buffers was identified as natural space in the final sample (by 2.0%).

### Statistical analysis

Statistical analyses were performed using SAS 9.2 software (SAS Institute Inc., Carry, NC). Key variables were described using conventional descriptive statistics. Multilevel logistic regression modeling was used to examine relationships between natural space and individual- and area-level covariates and positive emotional well-being, while also accounting for the clustered nature of the sample. The PROC GLIMMIX procedure was used to fit generalized linear models with a binomial distribution and a logit link. All models used a Newton–Raphson with ridging technique to aid convergence. Initially, a null model was fitted to examine area-level variance. Based on an empty logistic regression model, the intraclass correlation coefficient (ICC) was 2.85%, indicating that 2.85% of the total variance in positive emotional well-being was accounted for by the area level.

Models were built incrementally, as per existing precedents for multilevel modeling [61]: Model 1 controlled for all individual-level covariates (level 1), Model 2 simultaneously considered individual- and area-level (level 2) covariates, and Model 3 fitted all statistically significant individual- and area-level covariates with backwards elimination at p < 0.05. Covariates were removed based on descending order of significance in the model, and the consequences of their removal on the main effect estimates were checked as per a change in estimate approach; variables that caused a change in the main estimates of greater than 10% were kept in the final model. Interaction terms were created for those considered *a priori* and tested within the final model. However, no significant interactions by age (*p =* 0.29), gender (*p =* 0.50), ethnicity (*p =* 0.17), or urban/rural geographic location (*p =* 0.28) were observed. Models were also fitted with random slopes of the main exposure variable, but the fit was not improved from the model assuming fixed slopes. Therefore, all models in the analysis contained random intercepts only. Since the outcome of positive emotional well-being was common (>10%), odds ratio from regression models were converted to relative risks using Zhang and Yu’s method [62].

A stratified analysis was also conducted since the distributions of various natural space features were found to be different across rural, small city, and metropolitan areas (data not shown). Finally, a sensitivity analysis was conducted because not all student participants may have lived within the 5 km buffer of their school. Distance from school was calculated using GIS, and the regression analysis was repeated for those students who lived within a 5 km direct distance of their school (N = 9 271), as per their home postal codes.

### Human subjects

The HBSC holds ethics approval from the Queen’s University General Research Ethics Board (approval GEDUC: 430–9). Consent was received from school boards, schools, and parents/guardians prior to survey execution. At the student level, participation was based upon implicit or explicit consent, as per local school board requirements.

## Results

### Descriptive findings

Descriptions of the student population (N = 17 249) and participating schools (N = 317) are provided in Tables 1 and 2. Slightly higher proportions of girls were included compared to boys (by 5%) over a range of ages. The sample consisted mostly of Caucasian-identified students (71.0%), students from highly affluent families (57.7%), and students from small cities (41.1%). On average, 27.2% of the area within the 5 km buffers was comprised of natural space (17.4% green space and 9.8% blue space).

**Table 1:** Descriptive characteristics of student study population (N = 17 249)

**Variable**	**n**	**%**
*Individual-level*		
**Gender**		
Boys	8 196	47.5
Girls	9 053	52.5
**Age (years)**		
≤11	2 378	13.8
12	3 474	20.1
13	3 331	19.3
14	3 314	19.2
15	3 404	19.7
≥16	1 348	7.8
**Ethnicity**		
Caucasian	12 254	71.0
Aboriginal	1 085	6.3
East and Southeast Asian	1 032	6.0
South Asian	445	2.6
West Asian and Arab	212	1.2
Black	318	1.8
Other	578	3.4
Mixed	1 325	7.7
**Family affluence**		
Low (FAS 0–3)	1 305	7.6
Medium (FAS 4–5)	5 991	34.7
High (FAS 6–7)	9 953	57.7
**Neighborhood is safe for playing**		
1 (disagree/strongly disagree)	1 256	7.3
2 (neither agree/disagree)	2 904	16.8
3 (agree)	7 860	45.6
4 (strongly agree)	5 229	30.3
*Area-level*		
**Neighborhood rundown houses**		
Not a problem	12 989	75.3
Minor problem	3 187	18.5
Moderate/major problem	1 073	6.2
**Neighborhood litter/garbage**		
Not a problem	5 467	31.7
Minor problem	8 934	51.8
Moderate/major problem	2 848	16.5
**Neighborhood SES**		
Low (lowest tertile)	4 994	29.0
Medium	6 008	34.8
High (highest tertile)	6 247	36.2
**Urban/rural geographic location**		
Rural area (<10 000)	4 809	27.9
Small city (10 000–99 999)	7 085	41.1
Metropolitan area (≥100 000)	5 355	31.0

FAS, Family Affluence Scale.

SES, Socio-economic status.

**Table 2:** Descriptive characteristics of study schools (N = 317) according to area-level variables

**Variable**		
**Natural Environment**	**Mean**	**SD**
Total natural space	27.2%	22.9
Green space	17.4%	18.0
Blue space	9.8%	13.1
	**n**	**%**
**Neighborhood rundown houses**		
Not a problem	234	73.8
Minor problem	57	18.0
Moderate/major problem	26	8.2
**Neighborhood litter/garbage**		
Not a problem	103	32.5
Minor problem	163	51.4
Moderate/major problem	51	16.1
**Neighborhood SES**		
Low (lowest tertile)	97	30.5
Medium	109	34.4
High (highest tertile)	111	35.0
**Urban/rural geographic location**		
Rural area (<10 000)	90	28.4
Small city (10 000–99 999)	116	36.6
Metropolitan area (≥100 000)	111	35.0

SES, Socio-economic status.

As seen in Table 3, a slightly higher proportion of boys reported positive emotional well-being compared to girls (58.5% versus 51.6%). There were noticeable and statistically significant trends in the associations among positive emotional well-being and younger ages, higher family affluence, higher perceived neighborhood safety, lower neighborhood rundown houses, and lower neighborhood litter/garbage problems. Ethnicity was also significantly associated with positive emotional well-being, with the proportion of students reporting positive emotional well-being lowest among Aboriginal students (41.4%). Positive emotional well-being was not noticeably different across levels of neighborhood SES or urban/rural geographic location.

**Table 3:** Bivariate and multivariate analyses of association between total natural space and positive emotional well-being (N = 17 249)

	**Positive emotional well-being**		**Model PEW**^**a**^	**Model 1**^**b**^	**Model 2**^**c**^	**Model 3**^**d**^
	**n**	**%**	**RR (95% CI)**	**RR (95% CI)**	**RR (95% CI)**	**RR (95% CI)**
**Total natural space**						
1 (0.0% - 8.8%)	2 270	55.5	1.00	1.00	1.00	1.00
2 (8.8% - 24.3%)	2 347	52.3	0.94 (0.88-1.00)	0.99 (0.94-1.03)	0.98 (0.93-1.03)	0.99 (0.94-1.03)
3 (24.3% - 46.3%)	2 731	57.5	1.03 (0.98-1.09)	1.05 (1.00-1.10)	1.05 (1.00-1.10)	1.05 (1.00-1.10)
4 (46.3% - 95.0%)	2 118	54.0	1.00 (0.93-1.06)	1.01 (0.95-1.06)	1.02 (0.96-1.08)	1.01 (0.95-1.06)
*P trend*			*0.36*	*0.23*	*0.11*	*0.22*
*Individual-level*						
**Gender**						
Boys	4 795	58.5	1.00	1.00	1.00	1.00
Girls	4 671	51.6	0.88 (0.86-0.91)	0.88 (0.86-0.91)	0.88 (0.86-0.91)	0.88 (0.86-0.91)
*P value*			***<0.0001***	***<0.0001***	***<0.0001***	***<0.0001***
**Age**						
≤11	1 451	61.0	1.00	1.00	1.00	1.00
12	2 098	60.4	0.99 (0.95-1.04)	1.00 (0.95-1.04)	1.00 (0.95-1.04)	1.00 (0.95-1.04)
13	1 838	52.2	0.91 (0.86-0.95)	0.91 (0.86-0.96)	0.91 (0.86-0.96)	0.91 (0.87-0.96)
14	1 739	52.5	0.87 (0.82-0.92)	0.87 (0.83-0.92)	0.87 (0.83-0.92)	0.87 (0.83-0.93)
15	1 693	49.7	0.81 (0.77-0.86)	0.83 (0.78-0.87)	0.82 (0.77-0.87)	0.83 (0.77-0.87)
≥16	647	48.0	0.78 (0.72-0.84)	0.81 (0.75-0.87)	0.81 (0.75-0.87)	0.81 (0.75-0.87)
*P trend*			***<0.0001***	***<0.0001***	***<0.0001***	***<0.0001***
**Ethnicity**						
Caucasian	6 963	56.8	1.00	1.00	1.00	1.00
Aboriginal	449	41.4	0.75 (0.69-0.80)	0.80 (0.75-0.86)	0.81 (0.75-0.87)	0.81 (0.75-0.87)
East and Southeast Asian	511	49.5	0.87 (0.81-0.93)	0.94 (0.88-1.00)	0.94 (0.88-1.00)	0.94 (0.88-1.00)
South Asian	263	59.1	1.03 (0.94-1.12)	1.07 (0.98-1.15)	1.07 (0.98-1.16)	1.07 (0.99-1.16)
West Asian and Arab	120	56.6	1.01 (0.89-1.13)	1.08 (0.95-1.19)	1.08 (0.96-1.20)	1.08 (0.96-1.20)
Black	166	52.2	0.95 (0.85-1.05)	1.03 (0.93-1.13)	1.04 (0.93-1.14)	1.04 (0.94-1.14)
Other	304	52.6	0.93 (0.86-1.01)	0.97 (0.90-1.05)	0.98 (0.90-1.05)	0.98 (0.90-1.05)
Mixed	690	52.1	0.92 (0.87-0.97)	0.95 (0.90-1.00)	0.95 (0.90-1.01)	0.95 (0.90-1.01)
*P value*			***<0.0001***	***<0.0001***	***<0.0001***	***<0.0001***
**Family affluence**						
Low (FAS 0–3)	495	37.9	1.00	1.00	1.00	1.00
Medium (FAS 4–5)	2 875	48.0	1.25 (1.17-1.33)	1.22 (1.13-1.30)	1.22 (1.13-1.30)	1.21 (1.13-1.30)
High (FAS 6–7)	6 096	61.3	1.30 (1.26-1.34)	1.27 (1.23-1.31)	1.27 (1.23-1.31)	1.27 (1.23-1.31)
*P trend*			***<0.0001***	***<0.0001***	***<0.0001***	***<0.0001***
**Neighborhood is safe for playing**						
1 (disagree/strongly disagree)	583	46.4	1.00	1.00	1.00	1.00
2 (neither agree/disagree	1 358	46.8	1.01 (0.94-1.08)	1.02 (0.94-1.09)	1.02 (0.94-1.09)	1.02 (0.94-1.09)
3 (agree)	4 116	52.3	1.13 (1.07-1.20)	1.12 (1.05-1.18)	1.12 (1.05-1.18)	1.12 (1.05-1.18)
4 (strongly agree)	3 412	65.3	1.39 (1.33-1.45)	1.35 (1.28-1.41)	1.35 (1.28-1.41)	1.35 (1.28-1.41)
*P trend*			***<0.0001***	***<0.0001***	***<0.0001***	***<0.0001***
*Area-level*						
**Neighborhood rundown houses**						
Not a problem	7 286	56.1	1.00		1.00	1.00
Minor problem	1 666	52.3	0.92 (0.87-0.92)		0.96 (0.91-1.01)	0.96 (0.91-1.00)
Moderate/major problem	514	48.0	0.85 (0.77-0.85)		0.93 (0.85-1.04)	0.92 (0.85-1.00)
*P trend*			***<0.0001***		***0.005***	***0.009***
**Neighborhood litter/garbage**						
Not a problem	3 089	56.5	1.00		1.00	
Minor problem	4 932	55.2	0.97 (0.92-0.97)		1.00 (0.96-1.05)	
Moderate/major problem	1 445	50.7	0.87 (0.81-0.87)		0.96 (0.90-1.03)	
*P trend*			***0.0003***		*0.58*	
**Neighborhood SES**						
Low (lowest tertile)	2 735	54.8	1.00		1.00	
Medium	3 217	53.6	0.99 (0.94-0.99)		0.98 (0.94-1.03)	
High (highest tertile)	3 514	56.3	1.03 (0.98-1.03)		0.97 (0.92-1.02)	
*P trend*			*0.20*		*0.21*	
**Urban/rural geographic location**						
Rural area (<10 000)	2 655	55.2	1.00		1.00	
Small city (10 000–99 999)	3 951	55.8	1.02 (0.97-1.02)		0.99 (0.94-1.04)	
Metropolitan area (≥100 000)	2 860	53.4	0.98 (0.92-0.98)		1.01 (0.97-1.07)	
*P trend*			*0.37*		*0.51*	

The intraclass correlation coefficient is 2.85%.

^**a**^Model PEW: Bivariate models of positive emotional well-being and each covariate.

^**b**^Model 1: Multivariate model with individual-level covariates only.

^**c**^Model 2: Multivariate model with individual- and area-level covariates.

^**d**^Model 3: Multivariate model with individual- and area-level covariates significant at p < 0.05 in Model 2.

RR (95% CI), Risk ratio (95% Confidence Intervals).

FAS, Family Affluence Scale.

SES, Socio-economic status.

### Relation between natural space and emotional well-being

Table 3 also presents a hierarchical series of multivariate analyses. The effect estimates observed for the relationship between total natural space and positive emotional well-being in the final model were not statistically significant, were inconsistent, and no significant linear trends were present. Compared to students living in the lowest quartile of natural space, the strongest relationship existed for those who lived in the third quartile (RR: 1.05; 95% CI: 1.00-1.10), although the magnitude of effect was weak.

Throughout the model building process, individual-level variables were consistently associated with positive emotional well-being. For example, in the final multivariate model, girls were less likely to report positive emotional well-being compared to boys (RR: 0.88; 95% CI: 0.86-0.91). Older students reported lower emotional well-being compared to their younger counterparts (RR for students ≥16 years compared to those ≤11 years: 0.81; 95% CI: 0.75-0.87). Aboriginal students reported lower emotional well-being compared to Caucasian students (RR: 0.81; 95% CI: 0.75-0.87). Students from highly affluent families experienced higher emotional well-being compared to those from the least affluent families (RR: 1.27; 95% CI: 1.23-1.31). Reported high levels of perceived neighborhood safety were associated with positive emotional well-being (RR: 1.35; 95% CI: 1.28-1.41). High levels of perceived rundown houses were weakly associated with decreased emotional well-being (RR: 0.92; 95% CI: 0.85-1.00).

Table 4 summarizes the focal relationships between the three measures of natural space with positive emotional well-being. The results were adjusted for the same covariates as determined in Model 3 of Table 3. The pattern of effects across quartiles appeared similar for total natural space, green space, and blue space. There was a significant linear trend observed for the overall relationship with blue space (*p = 0.04)*, although the effects were weak. Results from a stratified analysis showed different effects across urban/rural geographic location (Table 4). Although effects were weak, stronger protective effects of total natural space and blue space were detected in small cities compared to rural and metropolitan areas.

**Table 4:** Multivariate subgroup analyses of associations between various natural space measures and positive emotional well-being (N = 17 249)

**Quartiles**	**Total natural space**	**Green space**	**Blue space**
	**RR (95% CI)**^**a**^	**RR (95% CI)**^**a**^	**RR (95% CI)**^**a**^
1	1.00	1.00	1.00
2	0.99 (0.94-1.03)	0.98 (0.93-1.03)	1.02 (0.97-1.07)
3	1.05 (1.00-1.10)	1.03 (0.98-1.08)	1.06 (1.01-1.11)
4	1.01 (0.95-1.06)	1.01 (0.96-1.06)	1.04 (0.99-1.09)
*P trend*	*0.22*	*0.34*	***0.04***
**By urban/rural geographic location**			
Rural area			
1	1.00	1.00	1.00
2	0.94 (0.85-1.03)	0.98 (0.87-1.05)	1.01 (0.91-1.11)
3	0.97 (0.88-1.06)	1.03 (0.81-1.06)	0.93 (0.82-1.04)
4	0.94 (0.83-1.05)	1.04 (0.83-1.08)	0.99 (0.91-1.07)
*P trend*	*0.30*	*0.24*	*0.58*
Small city			
1	1.00	1.00	1.00
2	1.11 (1.01-1.20)	1.05 (0.95-1.15)	1.11 (1.02-1.20)
3	1.16 (1.07-1.25)	1.10 (1.01-1.18)	1.15 (1.07-1.24)
4	1.12 (1.03-1.21)	1.07 (0.98-1.15)	1.14 (1.05-1.22)
*P trend*	***0.03***	*0.11*	***0.008***
Metropolitan area			
1	1.00	1.00	1.00
2	0.97 (0.88-1.05)	0.98 (0.87-1.08)	1.01 (0.93-1.09)
3	1.05 (0.96-1.14)	1.03 (0.92-1.13)	1.10 (1.02-1.18)
4	1.03 (0.91-1.14)	1.04 (0.92-1.15)	1.07 (0.96-1.18)
*P trend*	*0.26*	*0.23*	***0.02***

RR (95% CI), Risk ratio (95% Confidence Intervals).

^**a**^Adjusted for gender, age, ethnicity, family affluence, perceived neighborhood safety, and neighborhood rundown houses as determined in Model 3 of Table 3.

### Sensitivity analysis

The sensitivity analysis conducted among students known to live within 5 km of their school is presented in Table 5. Characteristics of this subsample (N = 9 271) were similar to those of the main study sample. There was a weak protective effect found in the third quartile of total natural space, compared to quartile one (RR: 1.11; 95% CI: 1:05–1.17), and there was a significant linear trend (*p = 0.04)*. The pattern of effects for green space was inconsistent, was not statistically significant, and no linear trend was detected. With regards to blue space, there was a weak protective effect with a significant linear trend, and this was similarly observed for small cities and metropolitan areas. Differences in effects across urban/rural geographic location can only be compared between small cities and metropolitan areas because no results were obtained for rural areas due to lack of convergence in model building. The strongest effects were detected for the relationship between green space and positive emotional well-being in small cities, although once again, effects were modestly weak and no significant trend was detected.

**Table 5:** Sensitivity analysis of selected students known to live within 5 km buffer (N = 9 271)

**Quartiles**	**Total natural space**	**Green space**	**Blue space**
	**RR (95% CI)**^**a**^	**RR (95% CI)**^**a**^	**RR (95% CI)**^**a**^
1	1.00	1.00	1.00
2	1.00 (0.94-1.06)	1.00 (0.93-1.06)	1.02 (0.96-1.08)
3	1.11 (1.05-1.17)	1.05 (0.99-1.11)	1.08 (1.01-1.14)
4	1.03 (0.96-1.09)	1.03 (0.96-1.09)	1.08 (1.02-1.14)
*P trend*	***0.04***	*0.19*	***0.003***
**By urban/rural geographic location**			
Rural area			
1	1.00	1.00	1.00
2	*-*	*-*	-
3	-	-	-
4	-	-	-
*P trend*	*-*	*-*	*-*
Small city			
1	1.00	1.00	1.00
2	0.98 (1.01-1.22)	1.08 (0.95-1.20)	1.10 (0.97-1.22)
3	1.12 (1.07-1.32)	1.14 (1.03-1.24)	1.15 (1.03-1.26)
4	1.11 (1.00-1.22)	1.06 (0.95-1.16)	1.19 (1.07-1.29)
*P trend*	*0.05*	*0.35*	***0.001***
Metropolitan area			
1	1.00	1.00	1.00
2	0.97 (0.87-1.08)	0.98 (0.85-1.11)	1.03 (0.93-1.14)
3	1.11 (0.99-1.22)	1.03 (0.90-1.16)	1.14 (1.03-1.25)
4	1.10 (0.95-1.24)	1.12 (0.97-1.26)	1.15 (1.01-1.29)
*P trend*	*0.06*	*0.07*	***0.004***

RR (95% CI), Risk ratio (95% Confidence Intervals).

^**a**^Adjusted for gender, age, ethnicity, family affluence, perceived neighborhood safety, and neighborhood rundown houses as determined in Model 3 of Table 3.

## Discussion

Our study findings suggest that, in general, public natural space is not strongly associated with positive emotional well-being in young people. The observed associations were weak and lacked consistency. Sub-analyses with green space and blue space and the sensitivity analysis also demonstrated weak associations and inconsistent trends. However, the findings in small cities suggest a small difference in positive emotional well-being based on the context of geographic location, such that youth in small cities may benefit more from natural space. The trend detected with blue space suggests a mechanistic difference between how blue and green spaces influence emotional well-being. Nonetheless, the weak effect estimates render these observations inconclusive.

The pattern seen in the effect estimates detected in quartile 3 suggests that moderate exposure to public natural space in one’s neighborhood environment may be most beneficial. A possible explanation for a lower protective effect in areas of higher exposure (i.e. quartile 4) may be linked to the composition of natural space. Different types of natural space may have differential importance based on quality and relevance to youth. For example, quartile 4 was made up of a greater proportion of wooded areas (see Additional file 2). These types of green space may not be maintained and/or conducive to activities similar to areas such as parks. This suggests that these areas may not be as relevant for emotional well-being among youth, if a relationship exists. Alternatively, the higher effect detected in quartile 3 may suggest a more optimal environmental composition for emotional well-being. For example, these neighborhoods contained more blue space, and thus the detected relationship may be explained by exposure to water.

Several personal factors were modestly associated with emotional well-being in young people. These findings were in general consistent with existing literature. Girls may be at greater risk of poor emotional well-being [8,31]; positive emotional well-being may decline with age through adolescence [8]; positive emotional well-being was associated with higher family affluence [19], higher levels of perceived neighborhood safety [55], and lower levels of perceived rundown houses in neighborhoods [63]. There were also differences across ethnicities, with positive emotional well-being particularly low among Aboriginal students [64], although some sample sizes were low for non-Caucasian students. These associations indicate the importance of individual factors on emotional well-being among youth. Further, the intraclass correlation coefficient (2.85%) supports that variation in emotional well-being was more accounted for by the individual level rather than the area level.

Although previous population studies have made conclusions for an association between nature and health, many studies found weak effect estimates, similar in magnitude to those detected in our study. Research from the Netherlands has found varying degrees of associations between green space and high perceived health (β: -0.009; SE: 0.003 [35]; β: 0.006; SE: 0.001 [27]), and low prevalence of mental health illnesses (OR range: 0.95-0.98) [31]. Similarly, a study from England showed that those from areas of higher green space reported lower rates of poor health (β: -0.021; *p* < 0.000; R^2^ = 0.8398) [28]. A study among Japanese seniors yielded weak odds ratio of 1.13-1.17 for a relationship between green space access and longevity [32].

Compared to studies that have found relationships between natural space and health, there are a number of possible explanations for the null findings of our study. First, studies, such as Sugiyama *et al.*’s [35], concluding strong associations have been observed in adults, and it is possible that the effect of natural space is different in young populations. Possible reasons for differences in effect between youth and adults may lie in variations in perceptions, usage, and interactions with natural space. Youth may not perceive natural space in the same way, and thus they may use and interact with natural space differently [65]. While adults may appreciate natural space for tranquility and reflection, youth may associate natural space more with play and socialization. Further, while adults may dictate their own decisions on where, when, and how to interact with natural space, youth may not have the same independent mobility because parents/guardians’ decisions may influence where they go and where they play [66,67]. Alternatively, for those youth with independent mobility, natural space may serve as gathering places for delinquent and antisocial behaviors instead of retreats for health promotion [67].

Second, the relationship between nature and emotional well-being may be context specific. As seen through the stratified analysis, effects were more pronounced for those in small cities. This finding was similar to Maas *et al.*’s observation that the relationship between green space and disease was strongest in slightly urban areas [31]. Young people may have a different relationship with public natural space based on the composition of their communities. Nature deficit disorder, a condition describing the decreasing use of natural space among youth because of increased electronic media and/or greater safety concerns surrounding young people being outside without supervision, is particularly high among urban youth [68-70]. In contrast, rural areas typically have an over-abundance of natural space, with few youth having low exposure. Therefore, the role of natural space may not be as important as other factors that affect emotional well-being. The lack of associations in rural areas in our study may be reflected by the lack of data for private and agricultural natural space, which account for a large proportion of the natural space in rural areas.

Further, our findings may be attributable to variations in geography, lifestyle, and/or culture that are specific to Canada. To our knowledge, no existing studies have assessed this relationship at a population level in Canada. Richardson *et al.* suggested that the high abundance and less spatial variation of green space in New Zealand, which is different compared to the Netherlands and England where much of the previous literature is based upon, accounted for the lack of association found between green space and mortality risk in their study [71]. Similarly, there is a different spatial composition in Canada compared to these countries. Although Canada has greater total land size and natural space area, the average natural space within the 5 km buffer measured in this study was lower than those measured in the Netherlands and England [27,28]. This may indicate that more of the natural space in Canada is located outside of the living neighborhood. The “car culture” [72] and tendency to drive may encourage Canadians to seek out faraway natural settings, and perhaps natural space close to home may not be as relevant. Additionally, climatic variations may play a role in how Canadians interact with the natural environment differently than those studied in other nations. For instance, patterns of usage of natural space would differ during the summer and winter months, and Canadians may have limited access to natural space due to winter conditions. This may have affected our ability to detect an effect as the HBSC was conducted during the fall, winter, and early spring.

### Strengths and limitations

Strengths of this study warrant comment. In a national analysis, we integrated health and spatial data to investigate the relationship between natural space and emotional well-being. This study intentionally focused this relationship in populations of young people. It is also one of the few population studies that have employed GIS techniques to obtain objective measures of natural space. Further, the study is well powered, and the multilevel modeling allowed for examination of effects at the individual and area levels. The analysis investigated and controlled for important covariates in the relationship of interest, which also addressed a methodological gap of previous research.

This study has some shortcomings. First, the cross-sectional design does not allow for confirmation of temporality, and subsequently, causality. Second, the measure of public natural space is limited because no data were available for privately owned natural space such as yards at the home and agricultural land. Therefore, the exposure measurement may be underestimated, particularly in rural areas. As well, this study was not able to consider the quality and usage of the natural space measured, which may be a critical part in the relationship between nature and emotional well-being. Third, the use of the 5 km radius buffer around schools may lead to misclassification of the natural space measures as this was used as a proxy for home neighborhoods. However, the findings in the sensitivity analysis suggest that this was not a major concern because results for those known to live within the buffer were similar to those in the overall study population.

### Implications

This type of research has the potential to inform the direction of health promotion strategies and urban planning decisions. Firstly, descriptive findings indicate that advocacy for policies and funding devoted to promotion of positive emotional well-being among youth is merited since only over half of young Canadians reported high levels. Exposure to public natural space appeared to have limited importance on positive emotional well-being of young people. There may be differences in this effect based on geographic context worthy of further consideration. Overall, focus should be placed upon more proximal variables such as individual factors and family affluence that showed stronger influences. This information is important to not only understand the health of this population, but may also help in the evaluation of current efforts that focus on emotional well-being among youth. Further, such knowledge may be useful in identifying vulnerable groups that require directed attention. Understanding factors that strongly influence emotional well-being may help to create more effective and specific public health programs and strategies.

Although findings of this study did not indicate a strong association between natural space and emotional well-being in young people, the potential health effects of natural space cannot be dismissed entirely. Natural space may, for instance, have different effects in adult populations, and it may impact other health outcomes such as physical activity that were not assessed in this study.

## Conclusion

This population study of Canadian youth did not provide strong evidence for the relationship between natural space and emotional well-being. The relation between nature and health may be context specific, and thus different for different geographic locations and for the Canadian population. Next steps in this field include studies examining the quality and usage of natural space and the role of context as determinants of well-being. Findings lend strong support that the main influences of emotional well-being among youth are personal factors. Efforts to promote positive emotional well-being in this age group must focus on the individual context as a priority.

## Abbreviations

HBSC: Health Behaviour In School-Aged Children; GIS: Geographic information system; SES: Socio-economic status; FAS: Family Affluence Scale; SAS: Statistical Analysis Software; RR (95% CI): Risk ratio (95% Confidence Intervals)

## Competing interests

The authors declare that they have no competing interests.

## Authors’ contributions

The conception and design of this study was a collaborative effort between all study authors. Access to and collection of data were led by WP and IJ. QH performed the statistical analysis and drafted the manuscript, with conceptual and editorial support from WP, WC, and IJ. All authors read and approved the final manuscript.

## Pre-publication history

The pre-publication history for this paper can be accessed here:

http://www.biomedcentral.com/1471-2458/13/407/prepub

## Supplementary Material

Additional file 1 Emotional well-being item on HBSC Survey. Click here for file

Additional file 2: Median proportions of natural space features by quartile of total natural space. Click here for file
