# Supplementary material for: Exposure to public natural space as a protective factor for emotional well-being among young people in Canada
Source: BMC Public Health. 2013 Apr 29;13:407. doi: 10.1186/1471-2458-13-407 (PMC3662164; doi:10.1186/1471-2458-13-407)
Supplement: Additional file 1 — Emotional well-being item on HBSC Survey. [file 1471-2458-13-407-S1.doc]

**Additional File 1:** Emotional well-being item on HBSC Survey.
