# Supplementary material for: Exposure to public natural space as a protective factor for emotional well-being among young people in Canada
Source: BMC Public Health. 2013 Apr 29;13:407. doi: 10.1186/1471-2458-13-407 (PMC3662164; doi:10.1186/1471-2458-13-407)
Supplement: Additional file 2: — Median proportions of natural space features by quartile of total natural space. [file 1471-2458-13-407-S2.doc]

**Additional File 2:** Median proportions of natural space features by quartile of total natural space.

| **Variable** | **Total Natural Space (Quartiles)** | | | |
| --- | --- | --- | --- | --- |
|  | **1 (0.0-8.8%)**  **%^a^** | **2 (8.8-24.3%)**  **%^a^** | **3 (24.3-46.3%)**  **%^a^** | **4 (46.3-95.0%)**  **%^a^** |
| Locals parks and sport fields | 0.13 | 2.26 | 0.78 | 0.27 |
| Provincial parks | 0.00 | 0.00 | 0.00 | 0.00 |
| Territorial parks | - | - | 0.00 | 0.00 |
| National parks | - | - | 0.00 | 0.00 |
| Other parks | 0.00 | 0.00 | 0.00 | 0.00 |
| Wooded areas | 0.56 | 5.04 | 9.46 | 50.40 |
| Campgrounds | 0.00 | 0.00 | 0.00 | 0.00 |
| Picnic areas | - | 0.00 | - | - |
| Golf courses and driving ranges | 0.00 | 0.72 | 0.45 | 0.00 |
| National wildlife areas | - | - | 0.00 | - |
| Bird migratory areas | 0.00 | 0.00 | 0.00 | 0.00 |
| Botanical gardens | - | 0.00 | 0.00 | 0.00 |
| Total green space | 1.91 | 9.53 | 15.05 | 51.36 |
| Total blue space (water bodies) | 0.62 | 3.08 | 14.04 | 9.61 |
| Total natural space | 3.16 | 13.93 | 34.08 | 68.86 |

^a^Percentage of total area in 5km buffer
